# Preliminary Evaluation of Muscle Fiber Composition in the Middle Gluteal Muscle in Race Mules and Mammoth Donkeys

**DOI:** 10.3390/ani16111640

**Published:** 2026-05-27

**Authors:** Raja Zabeeh Ullah Khan, Neil Gray, Francisco Javier Navas González, Amy K. McLean

**Affiliations:** 1 Animal Biology Graduate Group, University of California, Davis, CA 95616, USA; zabeehullah2395@gmail.com; 2 Equitarian Initiative, Steamboat Springs, CO 80477, USA; neil@ngraydvm.com; 3 Department of Genetics, University of Cordoba, 14014 Cordoba, Spain; 4 World Donkey Breeds Project, 14014 Cordoba, Spain; 5 Department of Animal Science, University of California, Davis, CA 95616, USA

**Keywords:** donkeys, equine muscle physiology, gluteus medius, mules, muscle morphology, muscle type

## Abstract

Research on mule and donkey muscle composition remains limited despite their global importance as working equids. The objective of this study is to identify Mammoth donkey jacks with higher percentage of fast twitch fibers for racing mule production. A total of 33 animals were biopsied; however, only 12 samples were suitable for analysis, including racing mules (*n* = 7) and male Mammoth donkeys (*n* = 5). Animals were sedated with detomidine (10 µg/kg body weight) and butorphanol (20 µg/kg body weight). Middle gluteal muscle biopsies were collected using a 6 mm Bergström biopsy needle at a site located 20 cm dorsocaudal to the tuber coxae at a 45° angle to the base of the tail. Collection depth was 7.5 cm in adult mules and 5 cm in donkeys. Samples were prepared aseptically, anesthetized subcutaneously with lidocaine hydrochloride, and frozen in liquid nitrogen. Histochemical analysis included myosin adenosine triphosphatase (ATPase) staining at pH 9.5, 4.6, and 4.3. Fibers were classified as Type I, Type IIA, or Type IIB, and CSA measurements were obtained using NIH ImageJ software. Statistical analysis included group contrasts, summarized as mean ± SD with 95% confidence intervals, while Bayesian ANOVA outputs were presented as exploratory evidence summaries. Type IIA fibers were greater in mules (47.84 ± 7.30%) than donkeys (38.47 ± 4.48%). Results suggest that differences in equid muscle architecture may be associated with variation in Type IIA fiber composition related to work or use.

## Simple Summary

Mules and donkeys are widely used as working equids for packing, harness work, and riding; however, limited information exists regarding their muscle composition. This study evaluated muscle fiber-type distribution and myofibril cross-sectional area in the gluteus medius muscle of racing mules and Mammoth donkeys using muscle biopsy samples. The objective was to provide preliminary data that may assist selective breeding programs aimed at optimizing performance for specific disciplines. Muscle fibers were classified as Type I (slow-twitch, high oxidative capacity), which are associated with endurance and slower gaits, and Type II fibers, including Type IIA (fast-twitch, oxidative-glycolytic) and Type IIB (fast-twitch, glycolytic), which support faster and more powerful movements over shorter durations. Of the 33 equids biopsied, 12 samples were suitable for analysis, including racing mules (*n* = 7) and Mammoth donkeys (*n* = 5). Analysis of muscle fiber composition demonstrated a greater proportion of Type IIA fibers in racing mules compared with Mammoth donkeys. However, no significant differences were identified between groups in muscle fiber cross-sectional area. These findings provide preliminary baseline information on muscle morphology in mules and donkeys and may support future research investigating equid physiology, performance, and selective breeding strategies.

## 1. Introduction

It is important to note that mules are the result of a breeding a male donkey (*Equus asinus*) to a female horse (*Equus caballus*) Historically, mules and donkeys are important animals for agriculture, goods transportation, and disaster relief work, especially in mountainous and high-elevation regions [1,2]. Despite rapid mechanization, mules and donkeys are still relevant in low- and middle-income countries as modes of the transportation of goods [3,4]. In the developed world, mules and donkeys find themselves adapting to new roles, like performances in racing and showing events [5]. The pre-existing and evolving roles of mules and donkeys require better breeding-related decision making and the knowledge to execute these decisions [6].

Equine performance may be limited based on skeletal muscle composition since it has a direct link with strength, speed and offset of fatigue [7]. Skeletal muscles are a heterogenous population of muscle fiber types whose architecture can vary with the function, location and metabolic demand of the muscle [8,9]. Skeletal muscle research focused on composition in horses has shown substantial variation within individuals, different breeds, and species, suggesting composition is related to endurance, sprinting, or strength-related activities [10,11,12,13,14]. These findings have contributed to the advancement and understanding of equine exercise physiology, neuromuscular and musculoskeletal pathology, nutritional management and breeding programs [10,15,16,17]. Evidence-based science has demonstrated horses bred for specific disciplines such as racing long distances and endurance have a higher proportion of slow twitch fibers, needing long-lasting energy and oxygen to fuel the muscles, compared to racing Quarter horses, who travel shorter distances requiring muscles to be quickly activated and fueled and present with more Type 2 fast twitch fibers (2A or 2B) compared to Thoroughbreds racing longer distances. However, comparable information on mules and donkeys remains limited across breeds of donkeys, and it is likely that few evidence-based breeding decisions have been made when breeding mules or hinnies.

With the increasing interest in rearing donkeys and mules as performance animals in activities such as racing, gymkhana, and endurance, understanding their muscle physiology has clinical, welfare and commercial implications [18]. Since mules are hybrids, they offer unique opportunities regarding the study and utilization of selective breeding through hybrid vigor (heterosis) [19]. This is because genetic expression can be affected by environmental factors which blur the role of genetics, but hybrids provide a good model for interspecific comparisons of gene expression profiles at the allelic level [20,21,22]. In equine breeding, these models could help frame why mules can surpass donkeys and horses in traits like endurance [19].

Recent molecular work is beginning to clarify the genetic basis of heterosis in equine hybrids using skeletal muscle from mules and reciprocal hybrids (hinnies) [19,20]. Gao et al. (2020) [19] reported widespread non-additive regulation in mule muscle, with a large proportion of differentially expressed genes showing high-parent dominance or over-dominance. Many loci also exhibit differential alternative splicing, suggesting regulatory mechanisms beyond expression changes alone. The fast-twitch troponin C (TNNC2) and sarcoplasmic reticulum Ca^2+^ release channel, essential for excitation–contraction coupling (RYR1), have been proposed as candidate mechanisms) showing dominant expression patterns in mules and substantially higher expression relative to horses, consistent with a potential contribution to endurance-related muscle function [19]. A second, complementary layer of genetic influence is the inheritance of mitochondrial DNA (mtDNA) solely through the maternal source [23]; because a mule is produced by a horse dam and donkey sire [2], it inherits mtDNA solely from the mare. Given the evolutionary divergence between horses and donkeys [24,25], maternal mitochondrial background plus gestational environment may influence muscle oxidative metabolism and energy-handling in the hybrid [23]. These findings suggest that mule muscle fibers can differ from their parent species quantitatively, qualitatively, and in terms of the biochemistry and functionality of sarcomeres.

Previous studies, mostly in horses and to a very limited extent in mules and donkeys, have shown differences in skeletal muscle composition that are also reflected as a mixed fiber distribution and adaptation in their endurance or sprint functions [14,26,27]. For example, Snow and Guy [27] reported breed- and species-associated differences in mATPase-defined fiber types in locomotor muscles, including the gluteus medius (GM), and Greene et al. [26] observed that mules had a higher proportion of oxidative fibers than Quarter horses in the GM. The gluteus medius is one of the most studied skeletal muscles in horses [28]. The GM lies dorsally and attaches close to the tuber coxae and sacral tuberosity of the ilium with a cranial insertion into the longissimus dorsi muscle [29]. This muscle is considered to have exceptional strength and size [30,31]. The GM muscle is used widely in studies for muscle biopsy sampling due to its safety, ease of access and importance in locomotion related to athletic capacity [31].

Fiber size and types in a muscle determine its functional output [32] in athletic performance, disease diagnosis, disease progression and recovery because fiber types are affected differently by varying stimuli [33,34]. Understanding muscle fiber morphology in mules requires integrating these genetic and physiological contexts. The aim of this study was to quantify the muscle fiber proportion of Type 1, Type 2A and Type 2B muscle fiber in the GM of mules and Mammoth donkeys. The broader objectives of this study were to potentially inform selective breeding decisions, clinical assessment and future research on equine muscle physiology in light of genetics, heterosis and endocrine functions.

## 2. Materials and Methods

**Figure 1:**
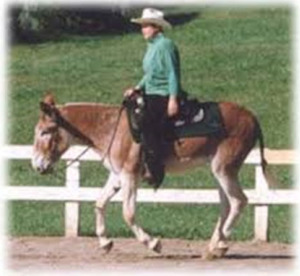
Mammoth donkey jack example A-Jacks El Gato, similar to donkeys sampled in this study with pedigrees and or performance records.

**Figure 2:**
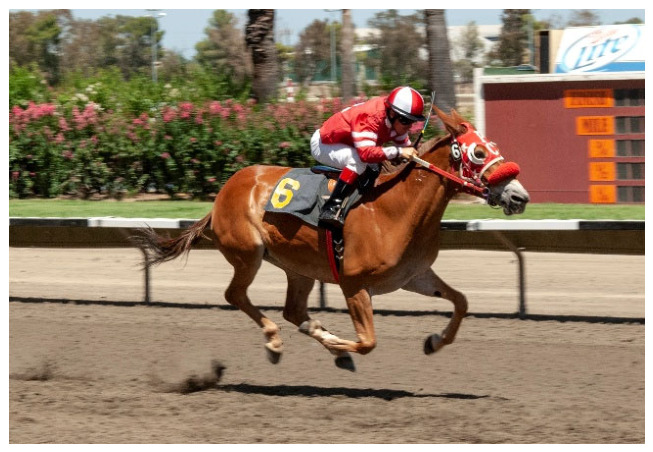
Award winning racing mule, Bar JF Red Ticket, used as part of the control for the study.

Animals: Thirty-three clinically healthy animals were sampled, including mules (*n* = 14) and Mammoth donkeys (*n* = 19). Prior to sedation and biopsy sampling, all animals underwent a veterinary physical examination and were deemed clinically healthy based on heart rate, respiratory rate, temperature, mucous membrane characteristics, capillary refill time, ocular and nasal discharge, and behavioral assessment. Mule ages ranged between 14 and 18 years, with seven females and seven males. The donkeys were between 6 and 17 years old, and four females and fifteen males were sampled (see Figure 1 and Figure 2 for examples of animals sampled). Mammoth donkeys were sampled for this study, and their muscle types were characterized and compared to race mules to learn more about skeletal muscle fibers’ relationship to the fibers desired for the fast twitch properties related to race mules. Sex, training status, and environmental conditions were not controlled and therefore represent study limitations. Additionally, most Mammoth jacks selected for this study had known pedigrees and were intended for potential future mule breeding based on the outcomes of the study. All the equid samples were obtained from within the United States of America with the owner’s consent and by a licensed veterinarian from the respective state of collection.

**Figure 3:**
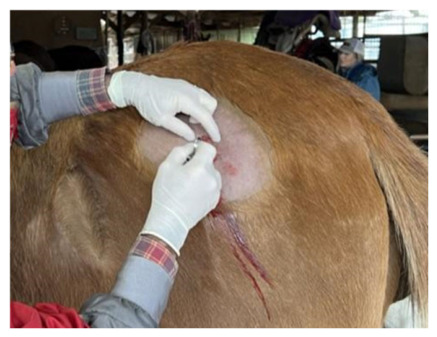
An incision was made at 20 cm dorsocaudal to tuber coxae at an angle of 45° to the base of the tail and then collected with a Bergstrom needle.

**Figure 4:**
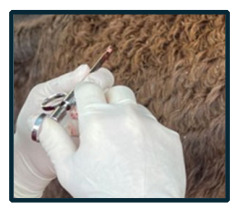
Bergstrom needle showing the sample taken from the GM muscle of a Mammoth donkey.

**Figure 5:**
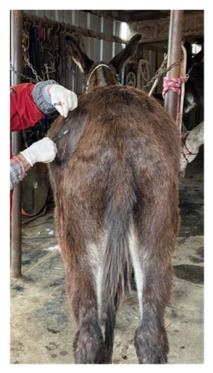
Sutures were placed after the sample was collected.

**Figure 6:**
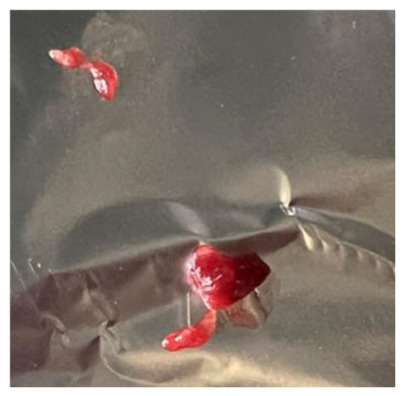
Sample shown after collection was labeled and then placed on dry ice, before being shipped immediately to the lab.

Muscle Biopsies: GM muscle was sampled according to the methods described by Lindholm and Piehl [35] with minor modifications. Animals were sedated with 10 µg/kg body weight of detomidine hydrochloride (Dormosedan^®^, Zoetis Inc., Parisppany, NJ, USA) and 20 µg/kg body weight of butorphanol tartrate (Torbugesic^®^, Zoetis Inc., Parsippany, NJ, USA), administered intravenously. Prior to sampling the area was prepared by clipping, shaving and scrubbing the area with disinfectant (e.g., povidone-iodine (Betadine^®^ Surgical Scrub, Aviro Health L.P., Stamford, CT, USA)). A local anesthetic (Lidocaine hydrochloride, VetOne^®^, Boise, ID, USA) was injected subcutaneously while avoiding the muscle. An incision was made at 20 cm dorsocaudal to tuber coxae at an angle of 45° to the base of the tail (refer to Figure 3). The depth of biopsy collection was 7.5 cm in adult mules and 5 cm in donkeys (in accordance with the UCD Neuromuscular Disease Guidelines). Samples were collected with a Bergstrom biopsy needle (Figure 3). The needle had an outer collection of 6 mms in diameter and a collection capacity of 250 mg of muscle, 5 cm long (see Figure 4). Following sample collection, the incision was closed (see Figure 5) and topical antibiotic ointment was applied. The samples were stored properly in dry ice and shipped overnight to the laboratory (Figure 6).

Histology and Fiber-Typing: Muscle samples were frozen in liquid nitrogen and stored at −80 °C until analysis. Cryosections at 10 µm were prepared and subjected to enzyme histochemistry as per Cotta et al. [36]. Myofibrillar adenosine triphosphatase (mATPase) staining was performed at pHs 4.3, 4.6, and 9.8, and hematoxylin–eosin (HE) staining was conducted in accordance with Dubowitz et al. [37] and Valberg [38].

Slides were examined using Leica light microscope (Leica Microsystems, Wetzlar, Germany) and images were captured using a mounted camera system and LAS X software (version 5.x) (Leica Microsystems). Random microscopic fields were selected and at least 100 fibers per fiber type were classified whenever possible. Image analysis for counting and measuring CSA for Type I, Type 2A and Type 2B muscle fibers was completed using ImageJ software (version 1.54, NIH ImageJ, Bethesda, MD, USA). Only areas without artifacts and myofibers with distinct borders were measured. Fibers were categorized based on the stain intensity between different fibers on different stains (Figure 1). For every slide of each sample, fiber-type proportions (Type 1, 2A and 2B) were calculated. CSAs were measured and computed using the semi-automatic features of ImageJ software. Samples were processed at University of California Davis Neuromuscular Disease Laboratory.

Statistical Analysis: Analyses were performed using IBM SPSS Statistics (27.0) (Bayesian ANOVA and Bayesian correlations) and XLSTAT (exploratory regularized discriminant analysis (rCDA) and CHAID classification tree). Bayesian ANOVA used Jeffreys–Zellner–Siow (JZS) priors, 95% credible intervals, Bayes Factors (BF10) and statistical significance was set at *p* < 0.05, to quantify evidence of group differences. The primary factor was species (donkey vs. mule). Multivariate classification was assessed using a CHAID classification tree to derive an interpretable species decision rule. For frequentist components, alpha was set at 0.05.

Exploratory Multivariable Analytical Approach and Statistical Justification: (expanded in Supplementary Material S1): Exploratory multivariable analyses (rCDA and CHAID) were used solely for hypothesis generation and should not be interpreted as predictive models [39,40,41,42,43].

## 3. Results

### 3.1. Sample Selection

**Figure 7:**
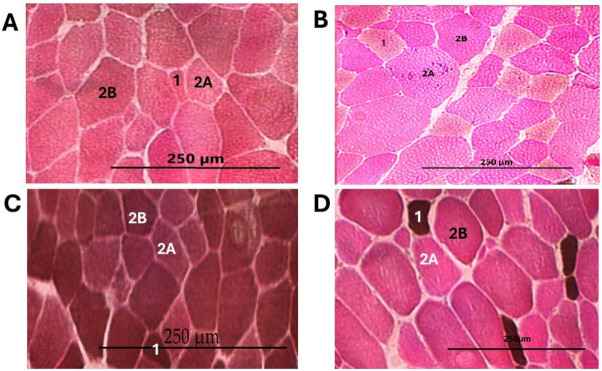
Representative mATPase staining of gluteus medius muscle fibers Types 1, 2A, and 2B in mules and donkeys. (**A**) Mule muscle fibers stained with mATPase at pH = 4.6. (**B**) Mule muscle fibers stained with mATPase at pH = 4.3. (**C**) Donkey muscle fibers stained at mATPase at pH = 4.6. (**D**) Donkey muscle fibers stained with mATPase at pH = 4.3.

Of the thirty-three samples collected, only seven mule and five Mammoth donkey muscle biopsy samples were suitable for quantitative fiber-type analysis (Figure 7). Samples were excluded from analysis due to one of two factors: insufficient muscle tissue collected during the biopsy process or sample damage during freezing and/or thawing in the laboratory. Mild non-specific myopathic alterations were observed in one of the donkeys and one mule, including internal nuclei and lack of mitochondria respectively.

### 3.2. Statistical Analysis

Type 2A proportion was higher in mules than donkeys (mean difference: 9.37 percentage points; 95% CI 1.76 to 16.98). Bayesian ANOVA gave BF10 = 16.7, providing moderate evidence of a group difference.

Bayesian ANOVA supported no species effect on Type 1, Type 2A, or Type 2B CSA (all *p* > 0.05; BF10 range 0.225–0.818, favoring the null) (Table 1).

The table shows myofiber-type composition (%), standard deviations (SD), ratio of Type 1 to Type 2 fibers, cross-sectional area (CSA), Bayes factor (BF) and frequentist ANOVA *p*-values; *p* > 0.05 is not significant. (*) marks significant *p*-value. Bayesian ANOVA did not support a difference in CSA between mules and donkeys for any fiber type. Extrapolated frequentist *p*-values > 0.05 and Bayes factors (BF10 range 0.225–0.818) provided evidence favoring the null model, consistent with the substantial overlap in estimated group distributions. The coefficient of variation (CV) for CSA differed between donkeys and mules across muscle fiber types. In Type 1 fibers, donkeys showed greater variability than mules (41.6% vs. 28.7%). In Type 2A fibers, variability was also higher in donkeys compared with mules (31.3% vs. 23.4%). Conversely, for Type 2B fibers, mules exhibited markedly greater variability than donkeys (46.8% vs. 31.7%).

### 3.3. Descriptive Statistics

**Figure 8:**
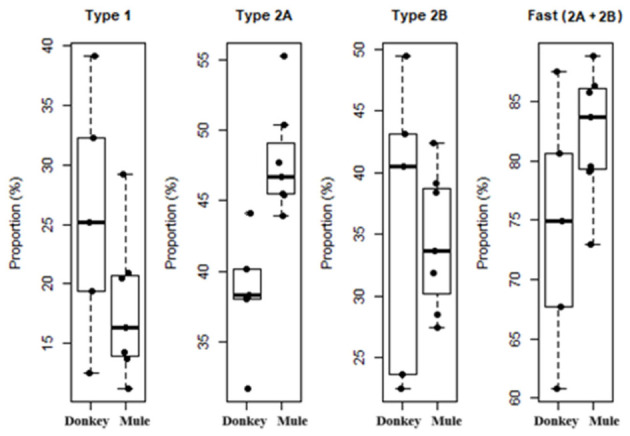
Boxplot showing frequency of mean percentage for each muscle fiber type (Type 1, Type 2A, Type 2B and fast twitch fibers combined (Type 2A + Type 2B)) when comparing Race mules and Mammoth donkeys.

Means (±SD) of fiber-type proportions and the ratio of Type 1 and Type 2 fibers (oxidative: glycolytic) were calculated (Table 1) and plotted (Figure 7). Means (±SD) of CSAs were calculated (Table 1) and plotted (Figure 8).

**Figure 9:**
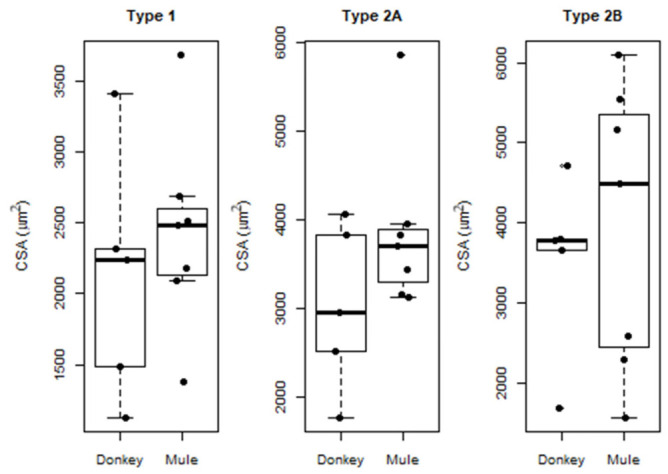
Boxplot showing mean CSAs for Type 1, Type 2A and Type 2B muscle fiber for the studied donkeys and mules.

Muscle Fiber CSA: Mean CSA values overlapped between species for all fiber types (Figure 9).

Correlation analyses demonstrated expected trade-offs among fiber-type proportions and positive covariation of fiber cross-sectional area (CSA) across fiber types. The strongest association among fiber-type frequencies was an inverse relationship between Type 1 and Type 2B proportions. CSA measurements were positively correlated, indicating that individuals with larger Type 1 fibers also tended to have larger Type 2A and Type 2B fibers.

Exploratory multivariable analyses suggested that Type 2A fiber proportion was the principal variable contributing to between-group differentiation. In the regularized canonical discriminant analysis (rCDA), Type 2A frequency consistently demonstrated the greatest contribution across model iterations. After reducing multicollinearity through selection of a four-variable model (variance inflation factors < 5), separation between groups remained modest (Wilks’ lambda, *p* = 0.072), with mules tending to exhibit higher scores along the canonical axis.

An exploratory CHAID classification tree identified a single split based on Type 2A fiber frequency (threshold = 44.09%), which separated most mules from donkeys within this dataset. Exploratory analyses suggested that the proportion of Type 2A fibers may contribute to between-group differentiation; however, given the limited sample size, these findings should be interpreted as exploratory and hypothesis-generating rather than inferential or predictive. Consequently, the observed classification patterns require validation in larger independent cohorts.

## 4. Discussion

This preliminary descriptive study reports gluteus medius fiber-type composition and fiber cross-sectional area (CSA) in race mules and Mammoth donkeys. Mules showed a higher mean Type 2A fiber proportion than donkeys, while Type 1 and Type 2B (proportions were similar between groups. Qualitative differences in fiber-type composition may distinguish mule from donkey muscle more effectively.

A previous study by Greene et al. [26], found a different distribution of fiber types than observed in this study for Type 1, 2A and 2B [26]. This difference could be related to several factors including the age distribution and work type of the populations studied, where our sample population was older mules of 14–18 years old used primarily for endurance and gymkhana events and Greene et al. [26] studied a population of pack mules that were between 6 and 12 years old, used for moving at slow speeds in mountainous areas carrying heavier amounts of weight on their backs and traveling only at a walk. Interestingly, this is in contradiction to the prevailing notion that older animals tend to have more Type 1 fiber due to atrophy of Type 2 fibers and motor unit remodeling [44,45,46]. Studies in horses have shown that horses bred and trained for racing had predominantly Type 2 muscles [14,27,46]. Other differences could be related to where the sample was taken. Samples in this study were collected 20 cm dorso-caudal to tuber coxae, yet Greene et al. [26] collected samples 10 cm dorso-caudal from the tuber coxae. Age, type of work/exercise and differences in sample collection may affect fiber distribution and results [12,36]. D’Angelis et al. [47] reported a higher distribution of Type 2A fibers than Type 1 and Type 2B in mules [47], which is consistent with the findings in this study. However, our results were different compared to their report in that they observed almost equal proportions of Type 1 and Type 2B fibers, whereas, in our case, proportions were different from each other [47]. Information on the type of mules used by D’Angelis et al. [47] and their age distribution is unavailable for discussion. Similarly, Snow and Guy [27] reported the muscle fiber composition of one donkey of unspecified breed. The percent frequency of Type 1, Type 2A and Type 2B fibers the in GM muscle of donkey(s) was comparable between our findings (Table 1) [27].

Type 2A fiber-type proportion (46.60 ± 7.30%) was higher in mules than in donkeys as in our study (38.47 ± 4.48) and as compared to the Type 2A fiber-type proportion for donkeys (38.2 ± 3.0%) reported by Snow and Guy [27]. Type 2B fiber-type proportion in our study is higher than those reported in different horse studies [48,49]. However, cross-study comparisons should be interpreted cautiously due to differences in cohorts, sampling, and fiber-typing methodology. This difference may suggest a potential heterosis effect contributing to the distinctive muscle fiber-type distribution observed in the mules in the present study. Transcriptomics works like that of Gao et al. (2020) reported certain genes (TNNC2 and RYR1) that express in a non-additive fashion in mules [19]. Even though these genes are not canonical fiber-identity regulators, as compared to Myosin heavy chain (MyHC) gene clusters or the Myostatin (MSTN) gene [14], future work pairing histochemical fiber-typing with transcriptomic profiling in the same muscle (gluteus medius) would be needed to test whether non-additive regulation in Ca^2+^ handling and contractile genes tracks directly with Type 2A fiber enrichment. Another genetic factor is the mule’s inheritance of maternal horse mitochondria. Because mitochondrial DNA is passed from the mare, mules carry horse-type oxidative properties in their muscle cells [19]. Horse mitochondria (from breeds selected for athletic performance such as Thoroughbreds or Quarter horses) may confer higher aerobic capacity in muscle tissue than donkey mitochondria, potentially contributing to the mule’s muscle metabolism and increasing oxidative (endurance) capacity beyond what would be expected from donkey genetics alone. This could result in the increase in observed Type 2A fiber frequency in our study, which was closer to a horse’s Type 2A fiber proportion than donkey’s. Mules used in this study were bred from Quarter horse or Thoroughbred mares.

Cross-sectional analysis suggested there was no difference between mules and donkeys. Therefore, Mammoth donkeys and mules may have a similar capacity to generate power and force from the gluteal muscle. The lack of any significant species difference in fiber CSA in our cohort was an interesting finding. Higher CSA is generally associated with higher-power-output muscles as thicker muscle fibers can pack more myofibrils in parallel, hence yielding more power [8]. This difference in size may be related to glycogen-storing abilities as well. Primary muscle differences between these species lie in fiber-type composition (e.g., Type 1 slow twitch or 2A or 2B fast twitch) rather than size. Knowledge surrounding muscle type may provide insight for breeders and trainers related to a mule’s performance (e.g., in terms of speed or load-carrying capacity), which is likely due to muscle quality (fiber-type makeup and metabolic capacity) more than to the quantity of muscle mass. This study may suggest that potential functional differences in mules are related to a higher proportion of Type 2A fibers that can both contract quickly and sustain aerobic metabolism.

Moreover, the endurance and metabolic potential of the muscles should also be considered in reference to species adaptations and genetics. Donkeys are evolutionarily adapted to survive harsh desert environments [50], and their natural responses to fear or pain are to freeze and/or fight, suggesting an anatomy adapted to slow twitch fibers or walking long distances for water or forage, leading to the higher proportion of fatigue-resistant muscle fibers observed in our study. Horses and zebras, in contrast, evolved in a grassy plains environment versus a desert like donkeys, where food and water were likely more readily available to refuel. When frightened, conserving energy was not as important, and they would flee [50]. These adaptive differences led to differences in anatomy, including muscle composition. Donkeys are generally adapted for moving slowly and horses are generally adapted for speed and power-oriented performance, with a higher number of fast twitch fibers and higher CSAs across all fiber types [27]. Mules, as hybrids, often inherit a blend of these traits from their parents, packaging the strength and athleticism of horses and hardiness and endurance of donkeys [51]. This blend of traits may be partially explained by the findings in this study, showing a higher number (or percentage) of Type 2A fibers in mules compared to donkeys. Type 2A fibers have both oxidative and glycolytic pathway properties and consistently higher CSAs across all fiber types in mules than donkeys but fewer than in Thoroughbred, Quarter horses, Standardbreds and Arabian horses, as observed by Aleman et al. [48].

In terms of limitations, differences in the methodology used to process samples have been reported. The Type 2B fibers found in donkeys, according to several studies, suggest that donkeys express myosin heavy chain (MHC) MHC-2B through the MYH4 gene [52,53]. This further suggests that mules may have MHC-2B genes because of their donkey parentage or hybrid isoform combinations. These and other potential biochemical differences also suggest that mule and donkey muscle biopsies should be processed differently to those of horses. One study standardized metachromatic mATPase histochemistry using toluidine blue and recommended treating the mule and donkey muscle biopsy samples at different pH levels, with acidic (4.5) and alkaline (10.5) preincubation steps to achieve the optimal differentiation of muscle fibers [54] and avoid the risk of the misclassification of different fibers. However, in this study, we used conventional staining rather than metachromatic staining and acid and base preincubation at pH 4.3, 4.6 and 9.8, respectively, due to the set laboratory protocols for horses.

As a large majority of the samples were lost during the lab processing stage, unfortunately, few studies can be used to compare muscle fiber composition and CSAs in mules and donkeys [26,27,47]. Moreover, similar to horses, there are breeds of donkeys which, like horses, may have different fiber compositions; therefore, additional studies comparing donkey breeds would be beneficial. Further research on different donkey breeds specific to muscle fiber composition and morphometry may provide more information related to their athletic or work abilities. This information is important for clinicians as well as donkey and mule breeders aiming to make clinical and breeding decisions based on fiber composition and size, which are related to heritable traits and may be affected by disease conditions, nutrition, environment and training [7,38,55].

This study should be considered preliminary. Quantitative fiber-typing was performed in only a subset of the available biopsies because some samples were excluded due to insufficient tissue, inconclusive staining, artifacts, or sample loss during freezing and thawing procedures. In addition, the number of readable fibers varied substantially among samples (approximately 66–400 fibers), which may have increased uncertainty in the estimation of both fiber-type proportions and cross-sectional areas (CSAs).

The primary aim of this study was to explore the theoretical potential for identifying “fast” donkeys based on muscle fiber composition. Accordingly, Mammoth donkey jacks from selected bloodlines and race mules with known racing histories and pedigrees were included in an attempt to identify jacks with a greater proportion of fast-twitch fibers that could potentially contribute to breeding mules with enhanced speed and racing performance. Future studies should include larger sample sizes and examine donkey breeds and mules selected for different types of work or athletic use.

In addition, future investigations into muscle fiber composition in donkeys and mules may benefit from incorporating sodium dodecyl sulfate–polyacrylamide gel electrophoresis (SDS-PAGE) and/or immunohistochemistry (IHC) to improve the characterization of myosin heavy chain isoform expression [56,57,58,59,60]. Compared with traditional histochemical staining and pH-based methods, these techniques provide greater specificity for distinguishing muscle fiber types, particularly hybrid fibers and subtle differences among Type I, Type IIa, and Type IIx fibers. SDS-PAGE enables the quantitative separation of myosin heavy chain isoforms, whereas IHC allows for spatial visualization of fiber-type distribution and morphology within muscle sections [56,57,58,59,60]. Together, these methods could improve the accuracy and repeatability of fiber classification in long-eared equids and facilitate more meaningful comparisons among donkeys, mules, and horses. Although these complementary techniques and validation of mammalian fiber-type–specific antibodies were not performed in the present study, their incorporation into future work could substantially strengthen comparative investigations of muscle physiology in equids.

To the authors’ knowledge, only one previous study in donkeys and one in mules have characterized GM muscle fiber composition, and no prior studies have reported fiber CSA measurements in these species. Overall, despite the limitations, this study contributes valuable preliminary reference information on gluteus medius muscle physiology in donkeys and mules and establishes an initial framework for future investigations in long-eared equids.

## 5. Conclusions

In conclusion, this preliminary descriptive study provides baseline information on gluteus medius (GM) muscle fiber-type frequencies and cross-sectional areas (CSAs) in racing mules and Mammoth donkeys. The findings expand the currently limited understanding of mule and donkey muscle physiology and support the need for additional research in long-eared equids. These data may assist in establishing species-appropriate reference expectations for the clinical interpretation of GM muscle biopsies and provide a framework for future studies investigating muscle adaptation, conditioning, performance, and comparative physiology in donkeys, mules, and other equids.

## Figures and Tables

**Table 1:** Gluteus medius myofiber composition (%) and CSA (µm^2^) descriptive and statistical analysis results in the studied donkeys and mules.

Group	*n*	Type 1 (% ± SD)	Type 2A (% ± SD)	Type 2B (% ± SD)	Ratio 1:2	CSA Type 1 (µm^2^ ± SD)	CSA Type 2A (µm^2^ ± SD)	CSA Type 2B (µm^2^ ± SD)
Donkey	5	25.67 ± 10.48	38.47 ± 4.48	35.84 ± 12.12	0.34 ± 0.28	2116 ± 881	3024 ± 946	3526 ± 1117
Mule	7	18.26 ± 5.10	47.84 ± 7.30	34.48 ± 6.90	0.22 ± 0.22	2432 ± 699	4015 ± 941	3812 ± 1784
*p*-value, BF10		*p =* 0.13, BF = 0.65	*p =* 0.003, BF = 16.6	*p =* 0.79, BF = 0.22	*p =* 0.30, BF = 0.41	*p =* 0.51, BF = 0.27	*p =* 0.10, BF = 0.82	*p =* 0.76, BF = 0.23

## Data Availability

The original contributions presented in this study are included in the article/Supplementary Material. Further inquiries can be directed to the corresponding author(s).

## Supplementary Materials

The following supporting information can be downloaded at: https://www.mdpi.com/article/10.3390/ani16111640/s1, Material S1. Exploratory Multivariable Analytical Approach and Statistical Justification.

## Abbreviations

The following abbreviations are used in this manuscript:

GM	Gluteus Medius
CSA	Cross-sectional area
mATPase	Myofibrillar adenosine triphosphatase
NIH	National Institutes of Health
