# Supplementary material for: Preliminary Evaluation of Muscle Fiber Composition in the Middle Gluteal Muscle in Race Mules and Mammoth Donkeys
Source: Animals (Basel). 2026 May 27;16(11):1640. doi: 10.3390/ani16111640 (PMC13255696; doi:10.3390/ani16111640)
Supplement: Supplementary file 1 [file animals-16-01640-s001.zip › animals-4296576-supplementary.pdf]

Supplementary Material S1. Exploratory Multivariable Analytical Approach and Statistical Justification.

Regularized canonical discriminant analysis (rCDA) and CHAID classification analyses were employed as exploratory and hypothesis-generating approaches to investigate potential multivariable patterns within this preliminary dataset rather than to establish inferential or predictive conclusions. The use of these methods was considered appropriate given the exploratory objectives of the study and the multivariate nature of muscle fiber composition data.

Regularized canonical discriminant analysis (rCDA) was selected because it is particularly suitable for datasets with small sample sizes, unequal group sizes, and multiple potentially correlated variables, all of which characterized the present study. Unlike conventional discriminant analysis, rCDA applies regularization (penalization) to covariance matrix estimation, reducing the risk of model instability and overfitting when the number of variables is relatively large compared with the number of observations. This approach improves the robustness and interpretability of classification analyses under limited-sample conditions and is therefore considered appropriate for exploratory investigations involving high-dimensional biological and histological data [39,40]. In the present study, rCDA was used as an exploratory analytical tool to identify potential patterns of separation between donkeys and mules while attempting to mitigate some of the statistical limitations associated with the reduced number of suitable biopsy samples.

Additionally, given the limited sample size, the CHAID analyses should be interpreted as exploratory and hypothesis-generating rather than definitive predictive models. CHAID (Chi-squared Automatic Interaction Detection) has previously been described as an exploratory classification and group-contrasting technique designed for categorical dependent variables and has been demonstrated using small illustrative datasets [41,42]. Although classification performance and model stability may decrease under small-sample conditions [43], CHAID remains useful for exploratory identification of potential variable interactions and preliminary classification tendencies. Accordingly, the analyses in the present study were used primarily to identify potential classification patterns and variable interactions while applying statistical approaches intended to improve robustness under reduced sample conditions. Therefore, the outputs of both rCDA and CHAID should be interpreted cautiously as exploratory observations intended to guide future research and hypothesis development in larger and more representative cohorts.
